# Therapeutic hypothermia for nonventricular fibrillation/ventricular tachycardia cardiac arrest

**DOI:** 10.1186/cc10888

**Published:** 2012-03-20

**Authors:** S Jog, D Patel, M Patel, R Kulkarni, N Chouthai

**Affiliations:** 1 Deenanath Mangeshkar Hospital and Research Centre, Pune, India; 2 Wayne State University, Detroit, MI, USA

## Introduction

Although efficacy of therapeutic hypothermia (TH) for cardiac arrest following ventricular tachycardia (VT)/ventricular fibrillation (VF) is a recommended therapy, the efficacy of TH for non-VF/VT cardiac arrest is still not well studied. We conducted a study to evaluate efficacy and outcomes of TH in non-VF/VT cardiac arrest patients in terms of survival and neurological outcome.

## Methods

TH was initiated with intravenous ice-cold saline and maintained with an external servo controlled cooling system (ESCCS); by Blanketrol II Hypo-Hyperthermia system (Cincinnati Sub-Zero Inc.) between 34 and 32°C for 24 hours. Gradual rewarming was also done with ESCCS. Non-VF/VT cardiac arrest patients with GCS ≤7 at 60 minutes of return of spontaneous circulation (ROSC) were enrolled. Standard hemodynamic monitoring and management was continued in all patients.

## Results

A total of 13 patients with average GCS of 3.4 at 1 hour after ROSC were enrolled in the study. Average time for ROSC was 16.5 minutes. Demographic and baseline variables were comparable amongst survivors and nonsurvivors except age (survivors 43 years and nonsurvivors 65 years). Average duration to achieve target temperature was 4.9 hours. Five out of 13 (38.46%) patients survived without any neurological deficit or cognitive dysfunction (Cerebral Performance Category - 1). Out of eight nonsurvivors, six died due to cardiogenic shock, one died due to refractory hypoxia and in one case relatives opted for withhold of aggressive care. Cardiac arrest was out of hospital in eight patients (three survivors and five nonsurvivors) and intra-hospital in five (two survivors and three nonsurvivors).

## Conclusion

TH may have beneficial effects in the neurological outcome of patients having non-VT/VF cardiac arrest. Additional controlled studies are warranted to establish efficacy of TH as a treatment for non-VT/VF cardiac arrest.

